# Late-glacial elevated dust deposition linked to westerly wind shifts in southern South America

**DOI:** 10.1038/srep11670

**Published:** 2015-07-01

**Authors:** Heleen Vanneste, François De Vleeschouwer, Antonio Martínez-Cortizas, Clemens von Scheffer, Natalia Piotrowska, Andrea Coronato, Gaël Le Roux

**Affiliations:** 1 Université de Toulouse, INP, UPS, EcoLab (Laboratoire Ecologie Fonctionnelle et Environnement), ENSAT, Avenue de l’Agrobiopole, 31326 Castanet Tolosan, France; 2 CNRS, EcoLab, 31326 Castanet Tolosan, France; 3 Departamento de Edafología y Química Agrícola, Facultad de Biología, Universidad de Santiago de Compostela, Campus Sur E-15706, Santiago de Compostela, Spain; 4 Department of Radioisotopes, Institute of Physics, Silesian University of Technology, Gliwice, Poland; 5 CONICET-CADIC, B. Houssay 200, 9410 Ushuaia, Tierra del Fuego, Argentina

## Abstract

Atmospheric dust loadings play a crucial role in the global climate system. Southern South America is a key dust source, however, dust deposition rates remain poorly quantified since the last glacial termination (~17 kyr ago), an important timeframe to anticipate future climate changes. Here we use isotope and element geochemistry in a peat archive from Tierra del Fuego, to reconstruct atmospheric dust fluxes and associated environmental and westerly wind changes for the past 16.2 kyr. Dust depositions were elevated during the Antarctic Cold Reversal (ACR) and second half of the Younger Dryas (YD) stadial, originating from the glacial Beagle Channel valley. This increase was most probably associated with a strengthening of the westerlies during both periods as dust source areas were already available before the onset of the dust peaks and remained present throughout. Congruent with glacier advances across Patagonia, this dust record indicates an overall strengthening of the wind belt during the ACR. On the other hand, we argue that the YD dust peak is linked to strong and poleward shifted westerlies. The close interplay between dust fluxes and climatic changes demonstrates that atmospheric circulation was essential in generating and sustaining present-day interglacial conditions.

The timing and nature of paleoclimatic changes in southern South America since the last glacial are still a matter of debate. A major issue is the reconstruction of changes in the intensity and latitudinal position of the southern westerly wind (SWW) belt as it exerts an important control on the Southern Hemisphere’s and global climate^1^. Fossil pollen, macrofossil records, glacier fluctuations, are among the several proxies used for this reconstruction^2^,^3^,^4^. Most of these indicators, however, are interpreted as proxies for precipitation but are in fact also influenced by temperature^1^,^4^. Furthermore they are based on present precipitation-SWW strength correlation, a relationship that is not straightforward and might have been different in the past^5^. On the other hand, dust deposition is a function of particle availability and transport^6^. The former is dependent on vegetation, ice and snow cover, while wind speed and gustiness are the main drivers of dust emissions^7^. Therefore, together with the available data on paleovegetation and ice cover in Patagonia^1^, paleodust records potentially provide unique information on wind strength and pattern changes. Patagonia is an important dust provider to the southwest Atlantic Ocean and the Antarctic Peninsula, particularly during glacial periods^8^,^9^. Since the Last Glacial Maximum (19–23 kyr ago^10^), however, dust accumulation rates dropped significantly in Antarctic ice cores^11^, corresponding with a significant glacier retreat in Patagonia^12^. Accordingly, this decline in dust has been associated with the formation of proglacial lakes due to glacier melt, trapping the sediments which were otherwise deposited on outwash plains, prone to mobilization^12^. To date, this hypothesis could not be verified because of a lack of paleodust records from Patagonia.

## Results

**Figure 1:**
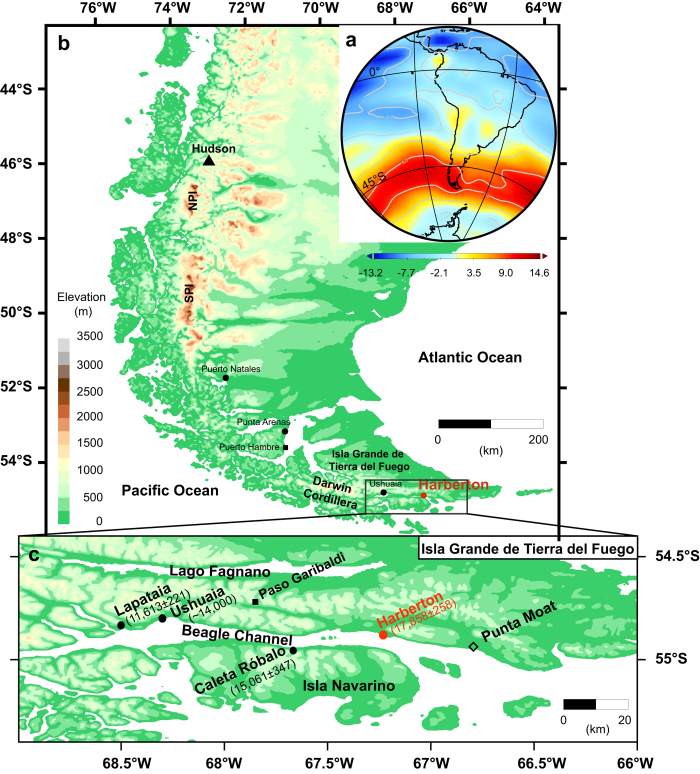
Modern climatology and Harberton bog location. **a**, Southern Hemisphere mean monthly zonal wind strength (m s^−1^) at 850 hPa based on NCEP/NCAR reanalysis data^56^, indicating present-day location of southern westerly wind belt. **b**, Topographic map of Patagonia (South America) and the location of the Harberton bog and paleoclimatic records discussed in text. NPI and SPI, respectively Northern and Southern Patagonian Ice Field. **c**, Topographic map of Beagle Channel and the locations of paleoclimatic records discussed in text and bogs for which the basal age has been measured (cal yr BP)^14^, indicating the timing of peat initiation. Punta Moat is the terminal moraine of the Last Glaciation^15^. Topographic maps were created in Surfer^®^ 8 using gridded topography xyz data extracted from the SRTM30_PLUS V10 global database^57^.

To investigate the dustiness of Tierra del Fuego during the late-glacial - Holocene, we sampled an ombrotrophic mire (referred to as Harberton bog; 54.9 °S, 67.2 °W) located on the south coast of Isla Grande de Tierra del Fuego along the Beagle Channel (Fig. 1). Ombrotrophic mires retrieve inorganic material solely via atmospheric deposition and therefore are excellent recorders of atmospheric dust content^13^. In addition, mires are abundant in southwest Patagonia^14^ due to high annual precipitation, showing high accumulation rates and are thus archives with potentially high-resolution chronologies. The base of the core is dated at 16.2 ± 0.2 cal kyr BP (Supplementary Table S1), indicating the approximate time of peatland initiation. This is slightly younger than earlier basal ages reported for this mire (17.8 cal kyr BP^15^), the latter indicating that Harberton was already ice-free by ~17.8 cal kyr BP as the result of deglaciation.

**Figure 2:**
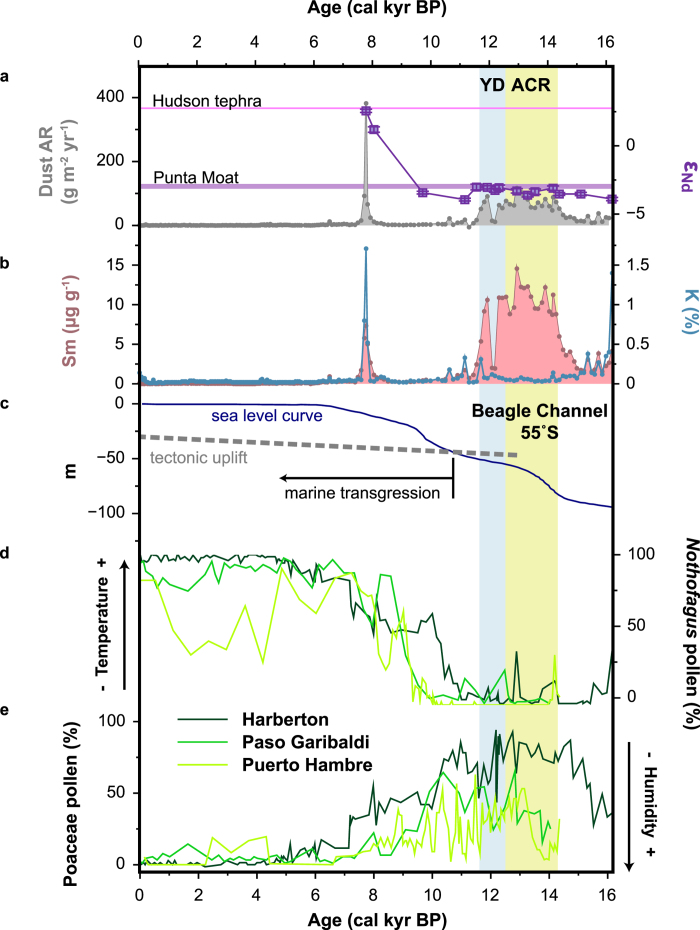
Paleoenvironmental data from the Southern Hemisphere. **a**, Dust accumulation rate (g m^−2^ yr^−1^) and neodymium isotopic composition (ε_Nd_) of the inorganic fraction of peat samples at the Harberton bog (54.9 °S, 67.2 °W; this study). **b**, Sm (ug g^−1^) and K (%) concentrations in bulk peat samples from Harberton bog (this study). **c**, Estimated eustatic sea level curve and local tectonic uplift (m) of the Beagle Channel area^19^. **d**,**e**, *Nothofagus* and Poaceae pollen records (%) from Harberton^58^, Paso Garibaldi^4^ and Puerto Hambre^20^ bogs (Tierra del Fuego and South-Patagonia). ACR = Antarctic Cold Reversal; YD = Younger Dryas.

The total dust accumulation rate shown in Fig. 2a is derived from elemental concentration-depth records, specifically from rare earth elements (REE) measured within the bulk peat samples at a resolution of ca. 100 yr. REE are considered to be conservative, insoluble and widely present in crustal rocks, hence they can be used as a quantitative indicator of the natural variation in mineral matter inputs to bogs. The Harberton record shows three periods of significant increases in atmospheric dust deposition (ADD) since the late-glacial: (1) from 14.8 to 12.2 cal kyr BP, (2) from 12.1 to 11.6 cal kyr BP and (3) from 7.8 to 7.7 cal kyr BP (Fig. 2a).

Apart from mineral dust, tephra layers are omnipresent in paleorecords from Patagonia^16^. Consequently, to extract the signal of atmospheric mineral dust deposition related to climate, we applied principal component analysis to the geochemical composition of the oldest section of the record (7.1 to 16.2 cal kyr BP), where the dust peaks occur. Two principal components explain 91% of the variance, which can be appointed to mineral dust (principal component 1, PC1) and volcanic ash (principal component 2, PC2). As 95% of the variance in Sm is due to PC1 and 93% in K to PC2 (see Supplementary Information Fig. S2), Sm and K are presented here as reference elements (i.e. consistent proxies) for respectively mineral dust (PC1) and volcanic ash (PC2). From the K and Sm profiles (Fig. 2b) it is clear that the oldest two dust peaks (at 14.8-12.2 and 12.1-11.6 cal kyr BP) are not associated with volcanic activity and therefore can be interpreted in terms of paleoclimate and environmental changes.

To further trace the origin of the atmospheric dust, we determined the neodymium isotopic composition of a selection of peat samples across the intervals with increased ADD (Fig. 2a and Table 1). Epsilon Nd values are centred at ca. –3.3 ± 0.2 (ranging from –3.0 ± 0.2 to –3.9 ± 0.1) with exception of two samples at 7.8 and 8 cal kyr BP, which have positive epsilon Nd values of respectively 2.6 ± 0.1 and 1.2 ± 0.2. The former is associated to a tephra layer of the Hudson volcano dated at 7.7 ± 0.3 cal kyr BP, for which an epsilon Nd value of 2.8 has been identified in a previous study^17^. The sample at 8 cal kyr BP with an epsilon Nd value of 1.2 ± 0.2 may represent a mixture of tephra material and atmospheric dust.

## Discussion

Glacier fluctuations have been suggested as an important dust generating process in South Patagonia^12^. Harberton area is a local drumlin field generated by an outlet glacier of the Darwin Cordillera ice cap^14^,^18^. This glacier covered the Beagle Channel and its surroundings during the Last Glacial Maximum, reaching as far east as Punta Moat^15^ (Fig. 1c). With the onset of the deglaciation the glacier retreated westwards (Fig. 1c), allowing the development of terrestrial and lacustrine environments in the ice-free valley until ca. 11 cal kyr BP, when the Beagle Channel was flooded by the sea (Fig. 2c)^19^. Accordingly, for the timespan of the mineral dust peaks in the Harberton record, the Beagle Channel was a potential dust source. Moreover the Nd isotopic signature of the last deglaciation terminal moraine at Punta Moat, −3.0 ± 0.1, is remarkably similar to the epsilon Nd values measured in the peat samples, −3.3 ± 0.2 (Fig. 2a), which is significantly more negative than the epsilon Nd values of any other known Patagonian dust sources (varying from −2.6 to 0.8^17^). Consequently, we suggest that the ice-free Beagle Channel was the main source of mineral dust for the south coast of Tierra del Fuego during the late-glacial.

Although the Beagle Channel became progressively ice-free west of Harberton since ~17.8 cal kyr BP^19^, we record two separate episodes of increased ADD, of which the eldest starts as early as 14.8 cal kyr BP. Furthermore, the regional vegetation cover was dominated by grassland and heatland between ~17 and 11 cal kyr BP (Fig. 2d,e), plant communities that thrive in a cold (i.e. colder than today) and dry environment^4^,^20^. Hence, despite the availability of sources and conditions for dust transport (low ice and vegetation cover, arid climate) since the onset of the mire formation (i.e. at 16.2 cal yr BP), dust deposition increased only significantly ca. 1.4 kyr later (i.e. at 14.8 cal kyr BP). This suggests a change in climate conditions 14.8 kyr ago, favouring dust mobilization. The timeframe of the first dust peak coincides with the Antarctic Cold Reversal (~14.1–12.8 cal kyr BP; Fig. 2b), a climate event known from Antarctic ice cores as a colder period^21^. Although the ACR is expressed in advances of outlet and alpine glaciers in respectively South Patagonia (51-52 °S)^22^ and the Fuegian Andes (54-55 °S)^23^, there are no reports of a glacier re-advances in the Beagle Channel^14^. On the other hand, proglacial lakes occupied deeper areas of the basin^19^. Accordingly, this data set shows that despite the presence of proglacial lakes, the Beagle Channel was still functioning as an important dust source for the region. Additional sampling may be needed to determine its impact, and that of other glacial valleys in Patagonia, on the environment at greater distances and ultimately on the global dust cycle.

After a brief episode of low dust fallout, a second dust peak was recorded from ~12.1 to 11.6 cal kyr BP, coinciding with the second half of the Younger Dryas (~12.9–11.7 cal kyr BP^24^, Fig. 2b). Whilst the YD was a cold and dry period in the Northern Hemisphere with an elevated atmospheric dust load^13^, there is still much debate on its existence and climatic nature in the Southern Hemisphere. Overall, outlet glaciers of the Southern Patagonian Ice Field diminished in size during the YD^25^,^26^. Pollen sequences from Harberton and neighbouring bogs however, point to a colder and drier steppe/tundra environment^4^,^20^. The intermittent dust record of Harberton is in contrast with the continuous low regional vegetation cover and the exposure of fine glaciogenic and glacial outwash sediments in the ice-free Beagle Channel valley during the late-glacial. Accordingly, dust transport may not have been limited by the availability of dust source areas, indicating that the aeolian transport capacity, i.e. the ability of the wind to transport sediment^27^, increased. This suggests that the westerly winds, which control the climate in Patagonia/Tierra del Fuego^28^, intensified during both periods. As Harberton bog is located on the leeside of the Andes it is subjected to Foehn winds. These downslope desiccating winds are more vigorous where strong westerlies cross the Andes therefore, outbalancing the westerly precipitation that comes in through the South Pacific^29^. Hence, the elevated ADD observed at Harberton is best explained by more vigorous westerly winds at the respective times.

**Figure 3:**
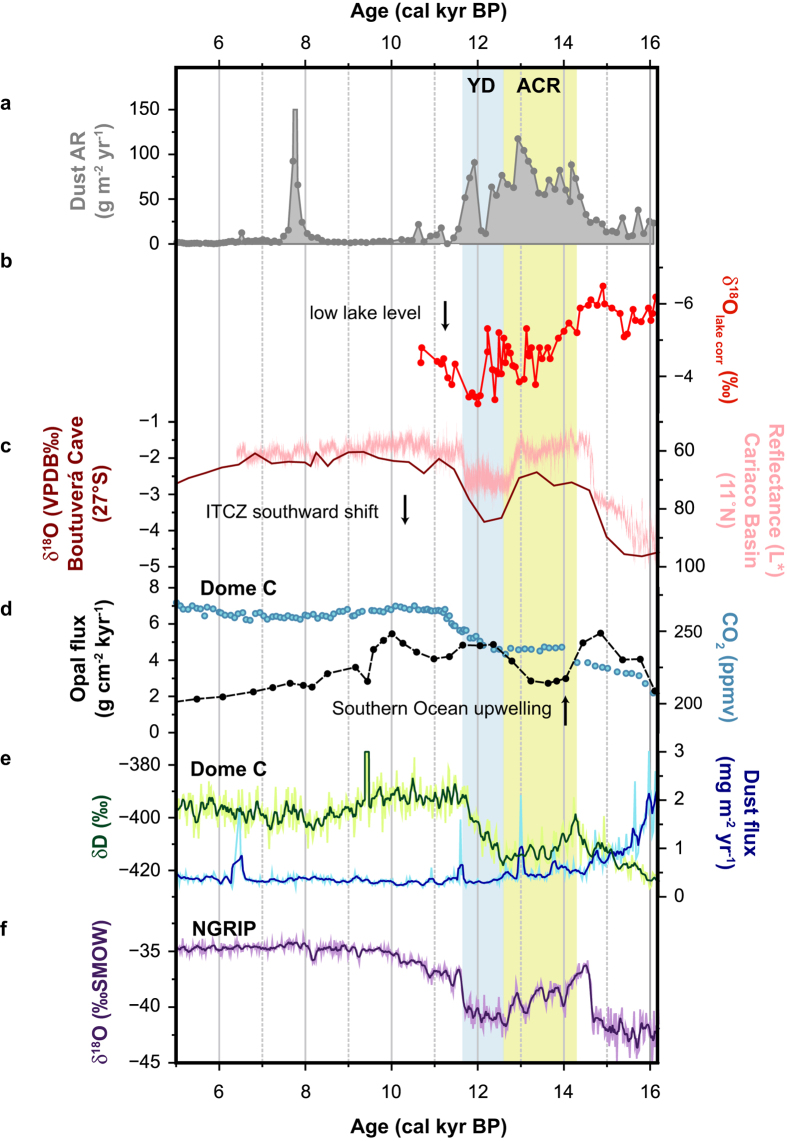
Proxy records of deglaciation during the last glacial termination. **a**, Dust accumulation rate (g m^−2^ yr^−1^) at the Harberton bog (54.9 °S, 67.2 °W; this study). **b**, corrected paleo lake-water oxygen isotopic composition (δ^18^O_lake corr_; ‰) from Laguna Potrok Aike (52 °S)^30^. **c**, speleothem δ^18^O values (VPDB ‰) from the Boutuverá Cave (South Brasil, 27 °S)^36^ and sediment reflectance record (L*) from the Cariaco Basin (11 °N)^35^, proxies for rainfall and hence the position of intertropical convergence zone (ITCZ). **d**, Opal flux (g cm^−2^ kyr^−1^) from a ocean sediment core in the South Atlantic, proxy for Southern Ocean upwelling^34^ and atmospheric CO_2_ concentrations (ppmv) from EDC on the EDC3 timescale^59^. **e**, EDC δD values (‰)^19^ on the EDC3 timescale, as a proxy of temperature and dust flux data (mg m^−2^ yr^−1^)^11^. **f**, δ^18^O values (‰SMOW) of NGRIP^24^. ACR = Antarctic Cold Reversal; YD = Younger Dryas.

In addition to the Harberton record, a number of paleoproxy records attest to an increased zonal flow during the ACR in both north (41 °S) and southwest (50–55 °S) Patagonia, indicating an overall strengthening of the westerlies^2^,^22^. On the contrary, to date, data on latitudinal shifts and intensity variations of the westerlies related to the YD climate event are scarce to non-existent in Patagonia and Tierra del Fuego^2^,^30^. Therefore, the Harberton dust record is of added value as it shows that westerlies were present and strong in Tierra del Fuego during the second half of the Northern Hemisphere YD. This is in agreement with an oxygen isotope record from Laguna Potrok Aike (52 °S; Fig. 3b): low oxygen isotopic values indicate low lake levels from 12.1 to 11.8 cal kyr BP due to increased evaporation induced by strong westerly winds^30^. The absence of similar observations further north in Patagonia, either indicates that the latitude, ~52 °S, represented the northern limit of the wind belt and/or the lack of appropriate wind proxies. For the ACR, glacier advances in the Andean Cordillera are linked to increased precipitation due to stronger westerlies^22^. Correspondingly, the onset of a glacial retreat at the Patagonian Ice Field (46°–51 °S) since the termination of the ACR till the Neoglacial^31^,^32^, could be interpreted as weakened westerlies during the YD, assuming precipitation was the main driver of glacier fluctuations^33^.

Based on this given data, we hypothesize that during the YD the wind belt most likely shifted southwards towards Antarctica or contracted. A southward displacement is in line with previous findings. The temperature asymmetry between the Northern and the Southern Hemispheres during the YD is believed to have driven the atmospheric intertropical convergence zone (ITCZ) towards the equator, pushing the westerlies southwards towards Antarctica^34^. The ITCZ displacement has been inferred by the occurrence of light coloured homogenous sediments in the Cariaco Basin (10 °N, Fig. 3c)^35^ and the low speleothem δ^18^O values measured in the Botuverá cave in southern Brazil (27 °S, Fig. 3c)^36^, generated during periods of respectively reduced and increased rainfall, i.e. when the ITCZ shifted southwards. Opal flux records from the Southern Ocean indicate enhanced westerly wind-driven upwelling during the YD (Fig. 3d), explaining the rise in atmospheric carbon dioxide concentrations in Antarctic ice cores (Fig. 3d)^34^. Accordingly, as we observe a climate signal in the Harberton dust record corresponding to the second half of the Northern Hemisphere YD, this dataset supports the view of an atmospheric mechanism^37^ besides the bipolar seesaw effect to explain the opposing behaviour between the hemispheres during the YD. The short and low ADD episode between the ACR and YD dust peak possibly indicates the timing of the reorganisation of the atmospheric circulation. This hypothesis will have to be tested in the future.

Regardless of the availability of dust in significant quantities, up to 120 g m^−2^ yr^−1^, in Tierra del Fuego during the ACR and YD, the majority did not reach Antarctica. Furthermore, besides the spike at 12.9 cal kyr BP in the Antarctic dust record (Fig. 3e), the latter differs significantly from the Harberton record (particularly from 16.2 to ~14.6 cal kyr BP). Does this imply that Tierra del Fuego, despite its close location, was not an important dust source for Antarctica at this time? Our dust record is in agreement with previous observations, which suggest that Australia and Antarctica have become more important relative to southern South American sources since 15 kyr, as a result of atmospheric circulation reorganisation^38^,^39^,^40^,^41^.

Our results fill an important geographical gap in the present-day global dust record. Furthermore, this data set demonstrates a great sensitivity of the dust cycle to relatively short climatic events (compared to glacial-interglacial transitions), which is most valuable for understanding and modelling dust dynamics.

## Methods

### Site Location

The Harberton bog (54.9 °S, 67.2 °W, 31 m a.s.l.) is one of the numerous mires located on the south coast of Isla Grande de Tierra del Fuego, along the Beagle Channel. Mean annual rainfall is around 600 mm yr^−1^ and mean annual temperature is 6 °C. The bog surface is dominated by *Sphagnum magellanicum* with a sparse cover of *Marsippospermum grandiflorum* and *Empetrum rubrum.* The present-day landscape of this region was mainly shaped by outlet glaciers of the Upper Pleistocene Darwin Cordillera ice cap: the Beagle Channel and Lake Fagnano glaciers besides alpine glaciers from the Fuegian Andes. Upon deglaciation, peatlands developed in small lakes and ponds in local drumlin fields, such as the Harberton area^15^,^42^, entrenched valleys and between moraines^14^.

### Sample collection

Peat cores were recovered from Harberton bog in 2012 by means of a stainless steel Wardenaar corer^43^ for the top meter, followed by a stainless steel Russian corer (50 cm length; 10 cm internal diameter) for the rest of the core. A second overlapping core was taken in case the initial one would show signs of disturbances caused by coring. This core was, however, not analysed as we did not detect any disturbances in the master core. All cores were photographed, described and packed in plastic film and PVC tubes to be stored in wooden boxes for shipment to France. At EcoLab, the cores were frozen at −20 °C, unpacked and sliced at 1 cm resolution using a stainless steel band saw. Subsequently, each slice was cleaned with MQ water, edges removed and subsampled for further analyses. The thickness of each slice was measured using a vernier caliper, to calculate the loss of material due to each cut. An average loss of 2 mm per cut was calculated (=[total core length (1050 cm) – cumulative sample thickness (891 cm)]/Nr of samples (739)) and accounted for when recalculating the mid-point depth of each sample. All the samples were stored at −20 °C. One till sample was collected in 2005 from the terminal moraine of the Beagle Channel glacier at Punta Moat, in a cliff formed due to the marine transgression and in a section of the pedo-sedimentary sequence (85–90 cm) where no pedogenetic transformations were evident. Before sampling, 10 cm of the outer face of the sediment were removed to avoid contamination or modification by present materials/processes. Detailed information on the geochemical and radiocarbon analyses as well as the age model is given in the Supplementary Information.

### Radiocarbon measurements and age model

Ten plant macrofossil samples were selected for radiocarbon analyses following established protocols^44^. All samples were prepared at the GADAM centre (Gliwice, Poland) where each sample was washed using the acid-alkali-acid extraction protocol (to remove carbonate, bacterial CO_2_ and humic/fulvic acid), dried, combusted and graphitised^45^. Radiocarbon concentrations were measured and ^14^C ages were calculated^46^ at the Rafter Radiocarbon Laboratory (Lower Hutt, New Zealand) and at DirectAMS Laboratory (Bothell, USA). Details of the dated material and results are given in Supplementary Table S1. The age-depth model was obtained using the Clam program^47^, which includes calibration of the ^14^C dates of the peat samples and a tephra layer of the Hudson volcano (dated 6850 ± 160 ^14^C yr BP^16^ at 682.6 cm depth) using the SHCal13 calibration curve^48^. The best fit was obtained using a smooth spline (Supplementary Fig. S1). Based on 10000 iterations, minimum and maximum ages for 2 sigma confidence interval were determined for each sample. Maximum likelihood ages were estimated based on the weighed average of all generated age-depth curves (Supplementary Table S2, excel file).

### Determination of major and trace element concentrations

A total of 205 bulk peat samples (at a ~5 cm resolution) were processed for element geochemistry. First, each sample was freeze-dried and powdered using an agate mortar. Subsequently, 100 mg of each sample was acid digested in Teflon beakers. The following digestion procedure was applied to each sample^49^: (1) a mixture of 0.5 ml HF and 2 ml 16 M HNO_3_ was added and left on the hotplate at 110 °C for 2 days; (2) 1 ml of H_2_0_2_ was added to react for 6 h at room temperature; (3) 2 ml of 16 M HNO_3_ was added and left at 90 °C for 2 days to finalize the digestion. After each step, samples were evaporated to dryness. Finally, the samples were dissolved in 2 ml of 35% HNO_3_, transferred into 15 ml polypropylene tubes (Falcon®), and further diluted with Milli-Q water up to 14 ml. Major elements (i.e. aluminium, titanium, magnesium and potassium) concentrations were determined by inductively coupled plasma optical emission spectroscopy (ICP-OES; IRIS Intrepid II). Trace elements (strontium, gallium, rubidium, zircon, cesium, scandium, lead, thorium, uranium, hafnium, rare earth elements) concentrations were determined by quadrupole inductively coupled plasma mass spectrometry (ICP-MS; Agilent Technologies 7500ce) at the *Observatoire Midi Pyrenees* (Toulouse, France). The ICP-MS was calibrated using a synthetic multi-element standard, which was run every 8 samples, while an In-Re solution was used as an internal standard. The analytical performance was assessed by analyzing 3 international certified reference materials: SRM1947 (peach leaves), SRM1515 (apple leaves) and GBW-07063 (bush branches and leaves). The results are reported in Supplementary Table S3. Measurements by ICP-OES of Mg, K and Ti were all within 10% of the certified values while the accuracy on the Al concentrations varied from 10 to 26%. The elements measured by ICP-MS were all within 15% of the certified values with exception of Sc (17%) and La (21%). The reproducibility of the digestion procedure, determined by repeat analyses of GBW-07063 (n = 10), SRM1947 (n = 7), SRM1515 (n = 3) and 6 peat samples (each n = 3), was generally better than 17%. The blanks for all elements considered here were <0.01 ppm.

### Neodymium isotopic composition of peat and till samples

Fifteen peat samples and 1 till sample were selected for neodymium (Nd) isotope analyses. To reduce complications associated with organic material during the separation process, 400 mg of bulk peat sample (100 mg for the till sample) was dry ashed in a furnace at 550 °C for 5 hours. Subsequently the samples were acid digested using a mixture of concentrated HNO_3_ and HF. The accuracy and reproducibility of the digestion procedure regarding Nd concentrations, determined by repeat analyses of the international certified standard GBW-07063 (n = 3), was better than 10%. Each sample was subsampled in order to make sure that a mass of 250 ng Nd was present in the sample that was loaded onto the columns.

Nd was separated from the matrix using a two-column ion-exchange technique. First, the rare earth elements (REE) were extracted from the sample using a cation exchange column (Bio-Rad poly-prep) packed with ~2 ml of Dowex AG50W-X8 resin (200–400 mesh). Then, Nd was isolated from the other REE by reversed phase chromatography^50^ using columns (0.4 cm inner diameter, 8 cm long) packed with Ln-Spec resin (50–100 mesh). Details on this separation procedure are given in Supplementary Table S4. Procedural blanks were consistently less than 2 pg g^−1^.

The Nd isotopic ratios were measured on a thermal ionisation mass spectrometer (TIMS) Finnigan MAT 261 (static mode) at *Observatoire Midi Pyrénées* (Toulouse, France). ^143^Nd/^144^Nd ratios were determined as the average of 150 ratios normalized to ^146^Nd/^144^Nd = 0.7219 to correct for instrumental-induced mass fractionation. The international standard La Jolla (^143^Nd/^144^Nd = 0.511858^51^) was analysed at every session to monitor instrumental drift. Measured values were 0.511851 ± 0.000006 (2σ, n = 3) which translates into an external precision of 0.1 ε_Nd_ units.

### Dust accumulation rate calculations

Dust AR (g m^−2^ yr^−1^) are calculated for each sample based on the REE concentrations measured in the peat samples using the following equations:


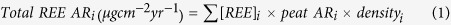



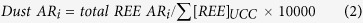


with ∑[REE]_i_ the of the concentrations (μg g^−1^) of the REE in sample i and ∑[REE]_UCC_ the sum of the REE concentrations in the upper continental crust (148 μg/g^52^). Peat AR (cm yr^−1^) and density (g cm^−3^) are given in Supplementary Table S2 (excel file). The density of each sample was determined by measuring the volume using a vernier caliper and subsequently weighing the sample after freeze drying it.

### Principal component analysis

Principal component analysis (PCA) was performed using SPSSStatistics 20 software using a varimax rotation to reduce dimensionality within the dataset. Based on the co-variance between the variables (i.e. chemical elements in this study), a number of components are extracted explaining the total variance within the dataset. In practice this means that the components will contain elements that show similar variation, in this case similar concentration-depth profiles. Before analysis all data were first log transformed (log10) and subsequently converted to z-scores to account for the compositional nature of the data. The latter are calculated as follows z = (X_i_−X_Avg_)/X_std_ with, X_i_ the concentration of element i, X_avg_ the average concentration of all samples for element i and X_std_ is the respective standard deviation. Both transformations rescale the data and hence open the closed system of concentrations^53^,^54^,^55^. A varimax rotation is an orthogonal rotation to optimize the loadings of variables in the components, i.e. to explain the variance in the dataset by more homogeneous components. PCA was only applied to the oldest section of the record (from 7.1 to 16.2 cal kyr BP) because we are interested in identifying the source of the dust peaks occurring in this section of the record. Secondly, the overall concentrations in the youngest part of the core are very low which would give a lot of noise to the data set and thus PCA outcome. The result of the PCA analysis is shown in Supplementary Fig. S2.

## Additional Information

**How to cite this article**: Vanneste, H. *et al.* Late-glacial elevated dust deposition linked to westerly wind shifts in southern South America. *Sci. Rep.* **5**, 11670; doi: 10.1038/srep11670 (2015).

## Supplementary Material

Supplementary Information

Supplementary Dataset S1

## Figures and Tables

**Table 1:** Neodymium isotopic signature of 15 peat samples from Harberton bog (54.9 °S, 67.2 °W) and 1 till sample from the terminal moraine at Punta Moat (55.0 °S, 66.8 °W).

Sample ID (depth)	^143^Nd/^144^Nd	±(2σ)	ε_Nd_	±(2σ)
*Peat samples*				
HAR12-PB01A-493 (683 cm)	0.512769	0.000006	2.56	0.12
HAR12-PB01A-502 (695 cm)	0.512699	0.000010	1.19	0.20
HAR12-PB01A-549 (760 cm)	0.512462	0.000005	−3.43	0.10
HAR12-PB01A-588 (814 cm)	0.512436	0.000005	−3.94	0.10
HAR12-PB01A-599 (829 cm)	0.512483	0.000011	−3.02	0.21
HAR12-PB01A-609 (843 cm)	0.512482	0.000006	−3.04	0.12
HAR12-PB01A-617 (854 cm)	0.512471	0.000007	−3.26	0.14
HAR12-PB01A-621 (859 cm)	0.512481	0.000009	−3.06	0.18
HAR12-PB01A-639 (884 cm)	0.512469	0.000008	−3.30	0.16
HAR12-PB01A-649 (899 cm)	0.512453	0.000007	−3.61	0.14
HAR12-PB01A-656 (910 cm)	0.512466	0.000010	−3.36	0.20
HAR12-PB01A-674 (935 cm)	0.512479	0.000008	−3.10	0.16
HAR12-PB01A-680 (944 cm)	0.512457	0.000005	−3.53	0.10
HAR12-PB01A-701 (974 cm)	0.512457	0.000006	−3.53	0.12
HAR12-PB01A-733 (1018 cm)	0.512438	0.000006	−3.90	0.12
*Till sample*				
Moat_I-30 (87.5 cm)	0.512486	0.000006	−2.97	0.12
