# Supplementary material for: Late-glacial elevated dust deposition linked to westerly wind shifts in southern South America
Source: Sci Rep. 2015 Jul 1;5:11670. doi: 10.1038/srep11670 (PMC4486931; doi:10.1038/srep11670)
Supplement: Supplementary Information [file srep11670-s1.doc]

Supplementary Information

**Late-glacial elevated dust deposition linked to westerly wind shifts in southern South America.**

Heleen Vanneste^1,2*^, François De Vleeschouwer^1,2^, Antonio Martinez-Cortizas^3^, Clemens von Scheffer^1^, Natalia Piotrowska^4^, Andrea Coronato^5^, Gaël Le Roux^1,2^

^1^Université de Toulouse, INP, UPS, EcoLab (Laboratoire Ecologie Fonctionnelle et Environnement), ENSAT, Avenue de l’Agrobiopole, 31326 Castanet Tolosan, France

^2^CNRS, EcoLab, 31326 Castanet Tolosan, France

^3^Departamento de Edafología y Química Agrícola, Facultad de Biología, Universidad de Santiago de Compostela, Campus Sur E-15706, Santiago de Compostela, Spain

^4^Department of Radioisotopes, Institute of Physics, Silesian University of Technology, Gliwice, Poland

^5^CONICET-CADIC, B. Houssay 200, 9410 Ushuaia, Tierra del Fuego, Argentina

*Corresponding author at: Laboratoire de Géologie de Lyon (LGL-TPE), Ecole Normale Supérieure de Lyon, 69364 Lyon, France.

E-mail address: [heleen.vanneste@ens-lyon.fr](mailto:heleen.vanneste@ens-lyon.fr) (H. Vanneste)

Pages: 5

Figures: 2

Tables: 4

**Table S1.** Sample composition and AMS radiocarbon dates for 10 peat samples from Harberton.

| **Sample Name** | **Sample** | **Sample** | **Age** | **Error** | **Depth** |
| --- | --- | --- | --- | --- | --- |
|  | **Composition** | **ID** | **(^14^C yr BP)** | **(yr)** | **(cm)** |
| HAR12-PB01A/47 | Sphagnum magellanicum branches, leaves & stems | GdA-2872 | 672 | 22 | 64.9 |
| HAR12-PB01A/193 | Sphagnum magellanicum leaves & stems | GdA-2874 | 3037 | 25 | 270.2 |
| HAR12-PB01A/262 | Sphagnum magellanicum leaves & stems | GdA-2875 | 3686 | 25 | 365.9 |
| HAR12-PB01A/332 | Sphagnum magellanicum leaves & stems | GdA-2876 | 4123 | 25 | 461.2 |
| HAR12-PB01A/421 | Unidentifiable Graminoid remains & Empetrum rubrum leaves | GdA-2877 | 5395 | 28 | 584.8 |
| HAR12-PB01A/499 | Unidentifiable Graminoid remains | GdA-2878 | 7136 | 36 | 690.8 |
| HAR12-PB01A/535 | Brown moss stems & leaves & charred Brown moss stems & leaves | GdA-2879 | 8300 | 34 | 740.2 |
| HAR12-PB01A/607 | Brown moss stems & leaves | GdA-2880 | 10257 | 42 | 840.5 |
| HAR12-PB01A/671 | Brown moss stems & leaves | GdA-2881 | 12202 | 51 | 931.1 |
| HAR12-PB01A/727 | Brown moss stems & leaves | GdA-2882 | 13335 | 59 | 1009.9 |

**Figure S1.** Age-depth model for the Harberton peat core. Maximum likelihood ages are expressed in calendar years Before Present (BP).


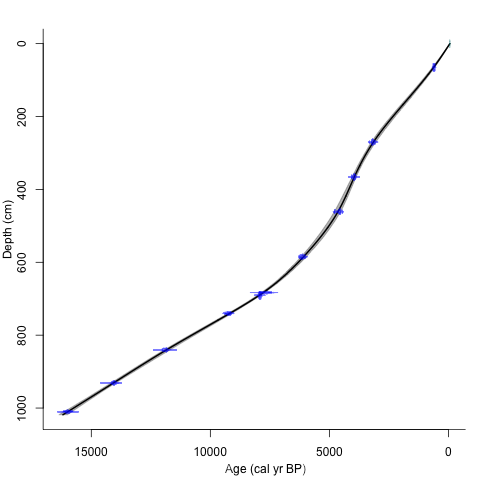


**Table S2.** Sample depths, maximum likelihood calendar ages, major and trace element concentrations for the Harberton core.

This table is given in a separate excel file.

**Table S3.** Accuracy and reproducibility on the peat sample analyses by ICP-OES and ICP-MS, determined by the analyses of certified reference materials GBW-07063, SRM1947 and SRM1515 (mean ± standard deviation; ug g^-1^).

| Elements | **GBW-07063** | | **SRM1947** | | **SRM1515** | |
| --- | --- | --- | --- | --- | --- | --- |
|  | **Measured**  **(n=10)** | **Certified^c^** | **Measured**  **(n=7)** | **Certified^c^** | **Measured**  **(n=3)** | **Certified^c^** |
|  |  |  |  |  |  |  |
| Mg^a^ | 4500±300 | 4800±400 | 3900±300 | 4320±80 | 2690±80 | 2710±80 |
| Al^a^ | 1800±200 | 2000±300 | 290±20 | 250±8 | 360±9 | 290±9 |
| K^a^ | 9100±800 | 9200±1000 | 22000±2000 | (24300) | 15600±300 | 16100±200 |
| Ti^a^ | 100±9 | 95±20 |  |  |  |  |
| Sc^b^ | 0.38±0.06 | 0.32±0.04 | 0.09±0.02 | (0.04) | 0.07±0.02 | (0.03) |
| Ga^b^ | 0.70±0.37 |  | 2.0±2.0 | (1) | 2.0±0.4 | (3) |
| Rb^b^ | 3.90±0.4 | 4.5±0.6 | 18.9±0.6 |  | 9.9±1 | (9) |
| Sr^b^ | 243±14 | 246±16 | 54±3 | (53) | 26±2 | 25±2 |
| Zr^b^ | 2.40±0.4 |  | 0.70±0.07 |  | 0.19±0.1 |  |
| Cs^b^ | 0.20±0.02 |  | 0.07±0.01 |  | 0.01±0 |  |
| La^b^ | 1.0±0.1 | 1.25±0.06 | 8.80±0.3 | (9) | 21±2 | (20) |
| Ce^b^ | 1.90±0.3 | 2.2±0.1 | 9.70±0.4 | (10) | 3.0±0.3 | (3) |
| Pr^b^ | 0.22±0.03 | (0.24) | 1.62±0.12 |  | 4.10±0.4 |  |
| Nd^b^ | 0.90±0.09 | 1±0.1 | 6.50±0.3 | (7) | 16.3±0.7 | (17) |
| Sm^b^ | 0.18±0.02 | 0.19±0.02 | 1.03±0.05 | (1) | 2.80±0.2 |  |
| Eu^b^ | 0.03±0.0 | 0.039±0.003 | 0.17±0.01 | (0.17) | 0.26±0.02 | (0.2) |
| Gd^b^ | 0.20±0.03 | (0.19) | 1.15±0.12 |  | 3.00±0.3 |  |
| Dy^b^ | 0.13±0.01 | (0.13) | 0.51±0.02 |  | 1.80±0.2 |  |
| Ho^b^ | 0.02±0.0 | (0.033) | 0.08±0.01 |  | 0.28±0.03 |  |
| Er^b^ | 0.07±0.01 |  | 0.20±0.01 |  | 0.55±0.04 |  |
| Tm^b^ | 0.01±0.0 |  | 0.02±0.0 |  | 0.05±0.01 |  |
| Yb^b^ | 0.06±0.01 | 0.063±0.009 | 0.12±0.01 | (0.2) | 0.19±0.02 | (0.3) |
| Lu^b^ | 0.01±0.0 | (0.011) | 0.02±0.0 |  | 0.02±0.0 |  |
| Hf^b^ | 0.08±0.02 | (0.15) | 0.02±0.0 |  | 0.02±0.01 |  |
| Pb^b^ | 45±3 | 47±3.0 | 0.78±0.08 | 0.87±0.03 | 0.42±0.02 | 0.47±0.02 |
| Th^b^ | 0.31±0.04 | 0.36±0.04 | 0.05±0.0 | (0.05) | 0.03±0.0 | (0.03) |
| U^b^ | 0.11±0.01 | (0.12) | 0.01±0.0 | (0.015) | 0.01±0.0 | (0.006) |

^a^Measured by ICP-OES; ^b^measured by ICP-MS; ^c^values within brackets are not certified but informative.

**Table S4.** Two-column ion-exchange procedure to extract Nd from peat samples

| **Cation columns** | | **Reverse phase columns** | |
| --- | --- | --- | --- |
| Step | Eluent (ml) | Step | Eluent (ml) |
| precleaning | 6M HCl (10) | Precleaning | 6M HCl (3) |
| Conditioning | 2.2M HCl (10) | Conditioning | 0.25M HCl (5) |
| Load sample | 2.2M HCl (2) | Load sample | 0.25M HCl (0.100) |
| Rinse sample tube | 2.2M HCl (2) | Rinse sample tube | 0.25M HCl (0.600) |
| Elute | 2.2M HCl (3) | Elute | 0.25M HCl (3) |
| Elute Ba | 2.5M HNO_3_ (10) | Collect Nd | 0.25M HCl (2.5) |
| Collect REE | 6M HCl (10) | Cleaning | 6M HCl (5) |
| Cleaning | 6M HCl (10) | Cleaning | 0.2M HCl (2) |
| Cleaning | H_2_O (10) |  |  |

**Figure S2.** The relative importance of each principal component in explaining the variance of considered elements. (The communality of an element is the total variance of the element explained by the extracted components; in the graph, the sections of the bar, black and grey, indicate the proportion of variance accounted for by the first and second component respectively).
